# Impact of Psychoeducation on Mental Health of Non-medical Students

**DOI:** 10.7759/cureus.77903

**Published:** 2025-01-24

**Authors:** Narayan K Ghorpade, Samson W Kamble

**Affiliations:** 1 Psychiatric Nursing, Bharati Vidyapeeth (Deemed to be University), Pune, IND

**Keywords:** child development psychology, mental health status, non-medical students, parenting, psychoeducation, public mental health, student mental health

## Abstract

Introduction: Mental health is an integral component of overall health and well-being, encompassing emotional, psychological, and social well-being. It influences cognition, perception, and behavior, and it determines how individuals handle stress, relate to others, and make choices. Mental health is essential at every stage of life, from childhood and adolescence through adulthood. The primary components of mental health include emotional well-being and psychological well-being. Emotional well-being refers to managing and expressing emotions effectively. It encompasses happiness, life satisfaction, and the ability to experience a range of emotions in a balanced manner. Whereas psychological well-being involves self-acceptance, personal growth, purpose in life, environmental mastery, autonomy, and other components. This research study examines the effectiveness of psychoeducation on the mental health status of students pursuing non-medical professional courses in colleges situated in Sangli city.

Objectives: The study aimed to evaluate the mental health levels of students enrolled in non-medical professional programs before and after receiving psychoeducation and compare these levels after the psychoeducational intervention.

Methodology: This study employed a pre-experimental pre-test and post-test research design. A purposive sampling technique was used to select the sample from the population, and 60 student participants of non-medical courses were included in this study. A pre-test was conducted followed by a psychoeducation session. A post-test was conducted after seven days to assess the effectiveness of psychoeducation sessions. Evaluations were included based on a standardized Mental Health Continuum-Short Form (MHC-SF) scale. The institutional ethical committee approved the study, and all guidelines, including consent and confidentiality, were strictly followed throughout the research.

Results: The pre-test mean score was 33.55 with a standard deviation of 11.3726, while the post-test mean score increased to 57.93 with a standard deviation of 7.0153. The p-value of 0.00001, significantly lower than the 0.05 threshold, indicated a highly significant difference between the pre-test and post-test scores. This suggested that the intervention had a substantial positive effect on the participants, with the results showing clear improvement in the measured variable. The findings' significance underscored the intervention's effectiveness in achieving meaningful changes in the participants' mental health status.

Conclusion: The results of the study indicate a statistically significant difference, with a p-value of 0.00001 at a 5% significance level. This supports the acceptance of the alternative hypothesis, demonstrating that there is a notable difference between the pre-test and post-test mean scores of mental health following the provision of psychoeducation to non-medical students. These findings suggest that psychoeducation has a meaningful positive impact, leading to significant improvement in the mental health of non-medical students in the post-test. Overall, this highlights the effectiveness of psychoeducation in enhancing the mental health status of students pursuing non-medical courses.

## Introduction

Mental health is essential to overall well-being, as defined by the World Health Organization (WHO). Adolescence (ages 10-19) is a critical period for social and emotional development, influencing long-term health. Enhancing personal coping abilities and support systems, both within oneself and through external networks, at this stage can help prevent future mental health challenges and foster overall well-being. While mental health problems often manifest in adolescence, with self-harm and suicide being the leading causes of death, interventions that strengthen family and social support can significantly reduce risk. Mental health issues, such as anxiety and depressive disorders, also contribute significantly to the non-fatal disease burden among adolescents, particularly among girls. As per WHO, up to 20% of adolescents experience mental health issues, with university students being particularly vulnerable due to the transitional phase of their lives [1]. Kumaraswamy's review (2013) examines the prevalence and impact of academic stress, anxiety, and depression among college students. The study highlights that these mental health challenges are common in students due to academic pressures, social adjustment issues, and personal expectations. The review explores how stress, anxiety, and depression can negatively affect students' cognitive, emotional, and physical well-being, leading to reduced academic performance, low self-esteem, and overall life dissatisfaction. Kumaraswamy emphasizes the need for universities and educational institutions to recognize these issues early and provide necessary support systems. Strategies such as stress management programs, counseling services, and promoting a balanced or healthy lifestyle are recommended to help students cope effectively with academic demands. The study concludes by stressing the importance of creating a supportive academic environment, where students are encouraged to seek help and develop healthy coping mechanisms to manage academic stress and mental health concerns, ultimately improving their academic success and well-being [2]. In India, the situation is similarly concerning, with numerous studies reporting high levels of stress and anxiety among college students [3]. Previous research has demonstrated that psychoeducation can effectively improve mental health outcomes by increasing awareness, reducing stigma, and promoting help-seeking behavior [4].

The study by Reavley and Jorm (2010) reviews the importance of prevention and early intervention strategies to improve mental health among higher education students. The research highlights that mental health issues such as depression, anxiety, and stress are common among university students, with significant implications for academic performance and overall well-being. The review emphasizes that early identification of mental health problems and the implementation of targeted interventions can significantly reduce the burden of these issues. The authors propose a range of prevention strategies, including psychoeducation, awareness campaigns, and accessible counseling services, which can help students develop resilience and coping mechanisms. Additionally, they suggest that institutions should adopt proactive approaches, such as mental health screenings and training for staff to recognize early signs of distress. The study concludes that a comprehensive approach to mental health in higher education, integrating prevention, early intervention, and ongoing support, is essential to ensure the well-being and academic success of students [5].

Adolescents, especially college-going students, suffer from both depression and anxiety at higher rates as the stressors/triggers are present in abundance. Emotional, behavioral, sexual, economic, academic, and social changes and efforts to discover one’s identity with psychosocial and maturation also occur. They go through a critical transitory period in their life, from adolescence to adulthood, making major life decisions. During this period, the mental health of university youth constitutes one of the important components of social health [6].

Strong social connections and supportive relationships are critical for mental health, providing a sense of security, belonging, and support. Mental health is a crucial aspect of overall well-being, particularly for undergraduate students who often face significant academic, social, and personal challenges. Psychoeducation, which involves providing individuals with information and resources about mental health, has emerged as an effective strategy for promoting mental well-being and reducing stigma [7]. Psychoeducation is the process of providing individuals with information and tools to better understand and manage their mental health, empowering them with knowledge to improve coping strategies, resilience, and overall well-being. In this study, we evaluate the mental health levels of students who are enrolled in non-medical professional programs, before and after receiving psychoeducational intervention, and compare these levels after the psychoeducational intervention.

## Materials and methods

This study aimed to evaluate the effectiveness of psychoeducation sessions using a quantitative research approach with a quasi-experimental pre-test and post-test design, conducted in selected colleges of engineering, management, and law in Sangli city, involving 60 non-medical students selected through a nonprobability purposive sampling method. Data collection utilized the standardized Mental Health Continuum-Short Form (MHC-SF) scale comprising 14 items related to mental health status (see Table 4 in Appendices). A pre-test was conducted with psychoeducation sessions and a post-test after seven days. The research proposal and data collection tool were reviewed and approved by the Institutional Ethical Committee (IEC) of Bharati Vidyapeeth Deemed to be University College of Nursing (BVDUCON), Sangli, under approval number EC/NEW/INST/2024/MH/0444, and prior permissions were secured from the concerned authorities. A total of 60 student participants, who met the inclusion criteria of students categorized as "languishing" or "moderately mentally ill" and enrolled in professional courses in law, management, and engineering, were informed about the study's purpose and protocol, and written informed consent was obtained. Students with "flourishing" mental health were excluded.

Participants faced no discomfort or risk, and confidentiality was ensured by coding the data collection tools. The intervention involved preparing a classroom with separate benches, a projector, and a comfortable platform, with formal intimation given to class coordinators to assemble students into convenient groups. Students were instructed to read and complete the consent form and the pre-test, which was followed by a 60-minute psychoeducation program, and the post-test was conducted seven days later.

Statistical analysis was performed using Microsoft 365 (Microsoft Corp., Redmond, US) and IBM SPSS Statistics Version 22 (IBM Corp., Armonk, US), employing descriptive statistics such as frequencies and percentages for qualitative data and mean with standard deviation for quantitative data. The paired t-test was used to assess the effectiveness of the psychoeducation program, while the chi-square test and Fisher’s exact test were applied to determine associations between demographic variables and knowledge, with a significance threshold set at p<0.05.

## Results

The instrument applied was a multidimensional tool designed to evaluate emotional, psychological, and social well-being. The collected data were systematically classified, organized, and analyzed to provide meaningful insights into the study's findings. The frequency and percentage distribution of participants based on selected demographic variables were examined to understand the characteristics of the participants. The analysis of pre-test and post-test levels of mental health status offered a detailed assessment of the participant's mental health before and after the psychoeducational intervention. Furthermore, a comparison between the pre-test and post-test levels of mental health status was conducted, highlighting the effectiveness of the psychoeducational program in improving the mental health of the participants

The majority of participants (63%) were aged between 18 and 20 years, followed by 35% aged 21-23 years, and only 2% aged 24-26 years. In terms of gender, 55% were male, and 45% were female. The distribution across professional courses was equal, with 33.33% of participants each from management, engineering, and law. All participants (100%) followed a semester-based course pattern, with no representation from annual patterns. Regarding the year of study, 27% of participants were in their fourth year, 23% each in their first and second years, 20% in their third year, and 7% in their fifth year. The semester distribution aligned with the year of study, with the largest proportion (27%) in semesters 7-8, followed by 23% each in semesters 1-2 and 3-4, 20% in semesters 5-6, and 7% in semesters 9-10. The demographic characteristics of the study participants are summarized in Table 1.

**Table 1:** Frequency and percentage distribution of participants (N=60) as per age, gender, course, course pattern, year of university study, and semester

No.	Demographic variables		Frequency	Percentage
1	Age in years	18-20	38	63%
		21-23	21	35%
		24-26	1	2%
2	Gender	Male	33	55%
		Female	27	45%
3	Course	Management	20	33.33%
		Engineering	20	33.33%
		Law	20	33.33%
4	Course pattern	Annual	0	0%
		Semester	60	100%
5	Year of university study	1	14	23%
		2	14	23%
		3	12	20%
		4	16	27%
		5	4	7%
6	Semester	01-Feb	14	23%
		03-Apr	14	23%
		05-Jun	12	20%
		07-Aug	16	27%
		09-Oct	4	7%

In the pre-test, none of the participants were categorized as flourishing, with the majority, 52 (87%), falling under the "moderately mentally ill" category and eight (13%) categorized as "languishing." However, the post-test results demonstrated a significant improvement in mental health status. Following the psychoeducational intervention, 57 (95%) participants were classified as "flourishing", with only three (5%) remaining in the "moderately mentally ill" category, and none in the "languishing" category. These findings underscore the effectiveness of the psychoeducational program in enhancing the mental health status of the participants. The analysis of the mental health status levels of the participants before and after the psychoeducational intervention is summarized in Table 2.

**Table 2:** Mental health status levels according to pre-test and post-test scores

	Level of mental health status					
	Flourishing		Moderately mentally ill		Languishing	
	f	%	f	%	f	%
Pre-test	0	0%	52	87%	8	13%
Post-test	57	95%	3	5%	0	0%

The mean mental health score in the pre-test was 33.55, with a standard deviation of 11.3726, indicating a lower level of mental health among participants before the intervention. In contrast, the post-test mean score significantly increased to 57.93, with a reduced standard deviation of 7.0153, reflecting a notable improvement in mental health status. The paired t-test yielded a t-value of 14.6711, with a p-value of 0.00001 well below the significance threshold of 0.05, indicating a statistically significant difference between pre-test and post-test scores. These results strongly suggest that the psychoeducation program was effective in improving the mental health status of the participants. The comparison of pre-test and post-test scores is presented in Table 3.

**Table 3:** Comparison between pre-test and post-test levels of mental health status

	Mean	SD	t-value	p-value	Inference
Pre-test	33.55	11.3726	14.6711	0.00001 (<0.05)	Significant
Post-test	57.93	7.0153			

## Discussion

This study aimed to evaluate the effectiveness of psychoeducation sessions on improving the mental health status of students in non-medical courses, with findings revealing significant improvements in participants' mental health after the intervention, thereby emphasizing the potential benefits of psychoeducation as a supportive mental health strategy. The demographic characteristics showed that most participants were aged 18-20 years (63%), with a nearly equal distribution of males (55%) and females (45%), representing students from management, engineering, and law courses equally, all following a semester-based curriculum, and predominantly in their fourth year of study or semesters 7-8 (27%), ensuring a balanced representation of students under various academic pressures.

Pre-test data indicated concerning mental health levels, with 87% of participants categorized as "moderately mentally ill" and 13% as "languishing," while none were "flourishing," highlighting the mental health challenges prevalent among students in professional courses, with existing research on academic and personal stressors. However, the psychoeducation program resulted in substantial improvements, with post-test results showing 95% of participants classified as "flourishing," 5% as "moderately mentally ill," and none as "languishing," demonstrating the intervention's effectiveness in promoting mental well-being.

Statistical analysis further validated these findings, with the paired t-test indicating a significant improvement in mental health status, as the pre-test mean score of 33.55 increased to a post-test mean score of 57.93, accompanied by a large t-value of 14.6711 and a p-value of 0.00001 (p<0.05), confirming the intervention's positive impact on mental health. These results underscore the importance of integrating psychoeducation into academic environments to address mental health challenges faced by students, suggesting its potential as both a preventive and therapeutic tool to enhance mental well-being. By using a biosocial and psychosocial model that addresses biological, psychological, and social factors, we can prevent and manage mental health challenges, particularly in high-stress academic environments.

The study showed that there was an improvement in the level of mental health status after giving psychoeducation. A similar study conducted by Srivastava and Panday highlighted psychoeducation as an effective treatment modality in mental health. Their results indicated that psychoeducation has emerged as a major therapeutic approach, enabling patients and their caregivers to become more skilled at coping with the manifold stresses caused by psychiatric disorders [8]. A study conducted by Ganesan et al. (2018) examined stress and coping strategies among undergraduate students. The findings suggested that targeted psychoeducation could help these students develop better stress management techniques, leading to improved mental health outcomes [9].

A similar study done by Negi et al. examined the psychological distress, stressors, and coping mechanisms of engineering students at the Indian Institute of Technology, Roorkee. Data collected from 76 M.Tech. and Ph.D. students revealed significant gender differences in stress, anxiety, and depression levels, with female students experiencing higher levels of psychological distress than their male counterparts. The use of the Depression Anxiety Stress Scale-21 (DASS-21) and informal interviews helped identify factors contributing to stress and the coping strategies employed. The findings suggested a need for targeted mental health support, especially for female students, to effectively address these challenges [10].

This study's findings are promising, offering valuable insights despite certain limitations. While the sample size (N=60) and single geographical location may narrow generalizability, it provides a focused perspective. Additionally, the pre-experimental design, despite lacking a control group, serves as a strong foundation for future research to build upon and refine the observed improvements.

## Conclusions

This study highlights the positive impact of psychoeducation sessions on improving the mental health of students enrolled in non-medical courses. Before the intervention, most participants experienced moderate mental health challenges, with many in the "languishing" category. Notably, no students were in the "flourishing" category during the pre-test, underscoring the need for mental health support in academic settings. After the psychoeducation program, 95% of students transitioned to the "flourishing" category, showing significant improvements in their mental well-being. Statistical analysis confirmed this effectiveness, with a marked increase in mean scores and a highly significant p-value. These findings emphasize the importance of integrating psychoeducation into academic curricula to promote mental health. While the study demonstrated success, its limitations such as a small sample size highlight the need for further research. Nonetheless, psychoeducation has proven to be an effective tool for fostering healthier and more resilient student communities.

## Appendices

**Table 4:** Mental Health Continuum-Short Form (MHC-SF) scale

No.	During the past week, how often did you feel...	Never (0)	Once or twice (1)	About once a week (2)	About 2 or 3 times a week (3)	Almost every day (4)	Everyday (5)
1	happy						
2	interested in life						
3	satisfied with life						
4	that you had something important to contribute to society						
5	that you belonged to a community (like a social group or your neighborhood)						
6	that our society is a good place, or is becoming a better place, for all people						
7	that people are basically good						
8	that is the way our society makes sense to you						
9	that you like most parts of your personality						
10	good at managing the responsibilities of your daily life						
11	that you had warm and trusting relationships with others						
12	that you had experiences that challenged you to grow and become a better person						
13	confident to think or express your own ideas and opinions						
14	that your life has a sense of direction or meaning to it						
